# Shepherds View of Large Carnivore Recovery in the Pyrenees, Spain

**DOI:** 10.3390/ani13132088

**Published:** 2023-06-23

**Authors:** José Ballarín, Alicia García-Serrano, Juan Herrero, Ramón Reiné

**Affiliations:** 1 Escuela Politécnica Superior, Universidad de Zaragoza, E-22071 Huesca, Spain; josecajigar@gmail.com (J.B.); herreroj@unizar.es (J.H.); 2 Ega Wildlife Consultants, E-50003 Zaragoza, Spain; aliciaega@gmail.com

**Keywords:** extensive sheep farming, mountain pastures, brown bear, wolf

## Abstract

**Simple Summary:**

The study was carried out in La Ribagorza County, Spain. We conducted 30 surveys on extensive sheep, *Ovis aries*, and goat, *Capra hircus*, farmers to discover their attitudes, perceptions, and knowledge of the current situation of this type of extensive farming and the conflicts that may arise between their activity and the presence of large carnivores such as the brown bear, *Ursus arctos*, or the grey wolf, *Canis lupus*. The coexistence of extensive livestock farming with the presence of large carnivores is one of the main challenges facing this sector.

**Abstract:**

The studied farms are small family businesses, and so, in more than half of the cases, their continuity is not guaranteed. Livestock management is typical of a mountain system, in which the animals graze throughout the year in cultivated fields, sown meadows, forests near the farms, and mountain pastures during the three summer months. The herds always have the constant surveillance of a shepherd. Farmers consider the current infrastructure present in mountain grasslands insufficient to facilitate the management and care of their herd. Their activity conflicts with various species of wildlife, such as the wild boar, *Sus scrofa*, roe deer, *Capreolus capreolus*, or griffon vulture, *Gyps fulvus*, and large carnivores such as the brown bear, *Ursus arctos*, or the grey wolf *Canis lupus*, despite all of them taking preventive measures to defend their herds from predators. The most widely used prevention measures are the presence of mastiff dogs, *Canis lupus familiaris*, next to the herds and the use of electric fencing to lock up livestock at night. Farmers reject the presence of bears and wolves in their area, considering it a real threat to the continuity of their economic activity, which presents a high degree of vulnerability.

## 1. Introduction

The pastoral areas of the Pyrenees have constituted a vital part of its landscape value and biological wealth, and its conservation is linked to the maintenance of grazing. In addition to pasture and soil improvements, these livestock practices are also essential for maintaining important species and habitats [1].

The presence of sheep herds is essential to preserve the current Pyrenean landscapes [2], both socially and environmentally, such as climate change mitigation, population fixation, rural development and land management.

In the Pyrenees, sheep, *Ovis aries*, numbers have decreased in recent years as they have suffered greatly from the new socioeconomic conditions [3]. This is despite the fact that this livestock activity has been prevalent for centuries and was the basis of the local economy in this mountain range, whose production system was based on grazing in mountain pastures during the summer and complementing it with pastures located in low areas, constituting the essence of the traditional transhumant system [4].

In recent years, brown bear, *Ursus arctos*, and grey wolf, *Canis lupus*, have been slowly recovering in Europe. This development presents substantial challenges for rural communities, especially in areas recolonized by large carnivores, where people have not lived with these species for decades [5]. The responses towards them range from admiration to hatred and the desire for extinction [6].

Protecting livestock herds is critical to deter predators and to prevent the development of problematic individuals that kill livestock preferentially, as poor protection can provide ample opportunity to learn predatory habits [7]. When there has been an attack, it is much more likely to be reproduced and so, its predictability should be used to reinforce prevention methods [8].

The current population of brown bears in the Pyrenees is the result of a repopulation of Balkan origin between 1996 and 2018 [9]. The wolf became extinct in the Pyrenees around the 1920s, however, thanks to the expansion of the Italic population, it has returned to colonize the Pyrenees in the last 20 years, although its reproduction has not been confirmed so far, and therefore there is no actual population [10].

This work aims to understand the attitudes, perceptions and knowledge of extensive livestock herders on the current situation of this type of extensive farming, their future, and the conflicts that may arise between their activity and the presence of large carnivores such as the brown bear and wolf.

## 2. Materials and Methods

The information collected in this study was obtained using a structured questionnaire, per Herrero et al. (2021) [11]. The population under study were the farmers who used the mountain pastures of the study area during the summer period to feed their herds. Before the start of the surveys, a list of candidates to participate in the work was drawn up. They shared the common characteristic of being farmers with a representative number of sheep in the Pyrenees context, specifically over 150 heads of sheep or domestic goats, *Capra hircus*.

The survey has been structured in blocks, from general to particular, describing first the farm characteristics (livestock species, size and structure of the herd, exploitation regime) followed by the pastoral practices in the summer pastures, the existing infrastructures and Protected Areas, the conflicts and interaction with other species, and ending with a block on the presence of the bear and the wolf and their opinion about it. The survey was undertaken by one of the research team(JB), who is also a farmer in the study area.

The types of questions used were: classification, binary, single or multiple choice, open, with jumps, frequency, intensity or Likert scale quotes. The average duration of each interview was 40 min. The interviews were previously arranged by telephone appointment with the farmers and were carried out in person on the farms owned by the interviewees. Before the beginning of the surveys, each participant was informed of the objective of the survey, its importance, who would carry it out, and the confidential nature of the study since personal references were eliminated from the generated databases and the questionnaires destroyed. The responses were anonymous, according to statistical secrecy and Spanish data protection laws (https://www.boe.es/eli/es/lo/2018/12/05/3/con (accessed on 23 May 2023)).

**Figure 1:**
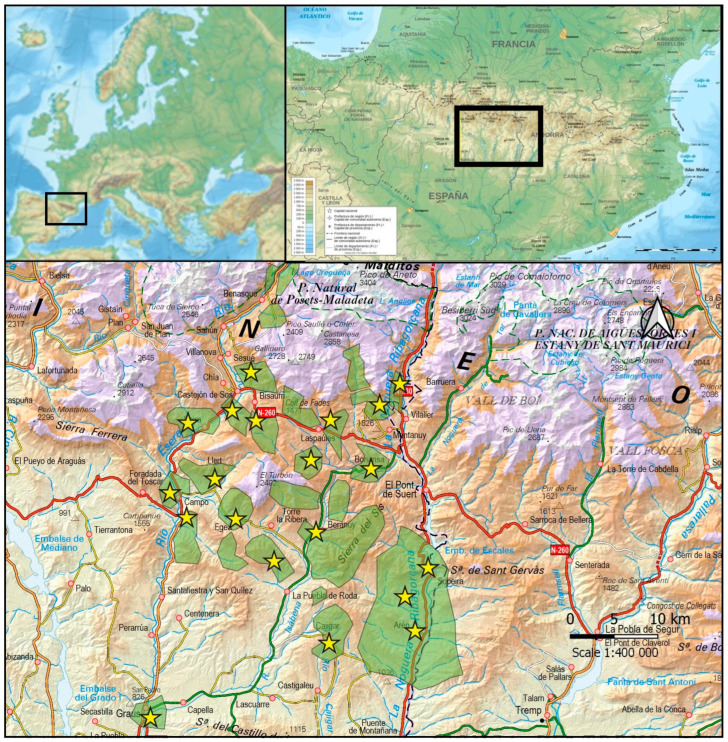
The farms (yellow stars) and pastures (green) in the study area.

The study area was La Ribagorza County, in central southern Pyrenees, Aragon region, Spain (Figure 1), totalling 2460 km^2^ and 34 municipalities. In 2020, there were 12,015 inhabitants (4.9 per km^2^) and 77,758 sheep [12]. Due to its tradition, structural conditions, abundance and the extension of its natural resources and its census volume, it is considered an eminent livestock region [13]. Farms are small, familiar, and traditional, and sheep graze in mountain areas [14].

38% of the county (925 km^2^) is part of its 16 Protected Areas, which belong to the European Natura 2000 Protected Areas network.

## 3. Results

### 3.1. Farms Characteristics

The 30 farms that have participated in the study add up to a total of 34,629 sheep, 44.5% of the total census of sheep in the county in 2020.

The farms were mostly family-type (97.7%, *n* = 30), and 2 were companies with 2 owners. Owners are mostly men (93.3%, *n* = 30), with a mean age of 46.9 years (SD = 13.2, *n* = 30). Although the most frequent age range was between 51 and 60 years, 37% were below 41 years of age.

Most were (86.7%, *n* = 30) full-time farmers; the rest combine their activity with trade and tourism. Most farms were inherited from previous generations (90.0%, *n* = 30): in fact, the majority were older than 25 years (76.6%, *n* = 30). A value of 33.3% of the cases (*n* = 30) do not have the possibility of generational relief due to a lack of descendants. Only 36.7% (*n* = 30) believe that their farm could have this family continuity. Newly created farms were the least numerous; only two were less than 10 years old (6.0%, *n* = 30).

The owner usually relies on the help of his family and receives the collaboration of one (46.6%, *n* = 30) or more (10.0%, *n* = 30) members. The usual external help is hiring one employee (23.3%, *n* = 30). One farm habitually hires 11 employees. Sporadic help from family origin is infrequent (10.0%, *n* = 30), although to help in the summer pastures hiring one employee is common (40.0%, *n* = 30).

When asked about the economic benefits of their farms over the last five years, no farmer considers that they have increased. Most (56.6%, *n* = 30) consider that they have decreased; less than 10% (41.2%, *n* = 17), between 10 and 20% (23.5%, *n* = 17) or even more than 20% (35.3%, *n* = 17). The remaining 43.4% (*n* = 30) consider that the benefits remain constant. A value of 26 of the 30 farmers work full-time. Although some of them complain about the low economic profitability of their farms, the amount of time they have to spend each day on their farm makes it impossible for them to combine it with another complementary job. Generational change is scarce; the decrease in benefits, the multiple problems faced by the sector, and the difficulty in many cases of reconciling the work of extensive shepherding and farming with family life mean that it is a less attractive trade for young people, even for the descendants of the farmers themselves.

Shepherds produce lamb meat on mixed farms of sheep with some secondary animals, mainly goats. In the analyzed sample (*n* = 30), this occurred on 63.3% of the farms, 16.6% were only sheep, 13.3% were sheep, goats and cattle, and 6% only goats. The average size of the sheep herd is 856.6 heads (SD = 448.3, *n* = 27). We have excluded from this average an abnormally large herd of 11,500 sheep and 400 goats belonging to the large farm mentioned previously. Taking this into account, the average number of heads of goats in mixed farms is 51.5 (SD = 27.7, *n* = 22) and that of cattle is 36.7 (SD = 40.3, *n* = 4). The total number of heads of livestock in the extensively managed farms studied is 34,629 sheep, 1424 goats, and 147 cows. The autochthonous and endangered Xisqueta sheep breed is dominant on the farms (85.7%, *n* = 28), the rest of the farms being crosses of other breeds. The breed of the goats is more variable; normally they are crosses of different origins (76.0%, *n* = 25), Pyrenean (20.0%, *n* = 25), Celtiberian White and Serrano Black (4.0%, *n* = 25). As for the cows, three farms have Alpine brown and one Limousin. The lamb and kid meat produced is not marketed under any protected designation of origin or geographical indication.

All sheep farms have a calving strategy of three lambings every two years and one annual calving of goats. Lambing is distributed throughout the year according to the management of each farm, less frequently in June and July. The mean number of months with lambing per farm was 5.1 (SD = 1.6, *n* = 28), being more frequent in autumn and winter. Most of the calving is simple, their average percentage was 67.5% (SD = 15.0, *n* = 28), and the rest is double 32.5% (SD = 15.0, *n* = 28), although it is noteworthy that four farms have higher percentages of double births than singles. The mean percentage of abortions in normal conditions is very low (2.7%, SD = 1.3, *n* = 28). The percentage of rams in the flock has a mean value of 2.3 (SD = 0.7, *n* = 28). The annual replacement rate is around 15% of the ewes in the flock, which compensates for the same percentages of estrangement and mortality.

### 3.2. Herd Feeding

The basis of the feeding is grazing in mountain pastures, forests, meadows, and cultivated fields of the valley bottoms, according to the time of the year. 70% of the farms (*n* = 30) only stall on specific days of bad weather, or when close to calving, the rest stall for some of the winter months (3.3% 1 month, 10% 2 months, 13.3% 3 months and 3.3% 4 months). In winter, feeding is based on dry hay supplemented with corn grain, *Zea mays*, and barley grain, *Hordeum vulgare*, depending on the farm. During the intermediate seasons, spring and autumn, livestock use cultivated fields (fodder monocultures such as alfalfa, *Medicago sativa*, sainfoin, *Onobrychis viciifolia*, vetch, *Vicia sativa*, or Italian ryegrass, *Lolium multiflorum*), sown meadows (with different mixtures of grasses and legumes), forest pastures near the farms dominated by holm oak, *Quercus ilex*, white oak, *Quercus humilis*, Austrian pine, *Pinus nigra*, and Scots pine, *Pinus sylvestris*, and intermediate pastures of the *Festuco-Brometea* phytosociological class. The daily grazing lasts 6–9 h, and the animals sleep on the farm (93.3%), although there are two cases (6.6%) of herds that are in the field all day. With the arrival of summer, 83.3% of the herds move to the mountain summer pastures, made up of various herbaceous communities included in *Festuco-Brometea*, *Caricetea curvulae*, *Festuco hystricis-Ononidetea striatae*, *Carici-Kobresietea*, and *Nardetea strictae* phytosociological classes. Herds remain for an average of 3.2 months in the mountain pastures (SD = 0.82, *n* = 25). The rest continue to graze in forest and scrub areas close to the farms.

### 3.3. Pastoral Use in Summer Ranges

Herds are taken to the mountain pastures at the beginning of the summer, covering an average of 40.2 km on foot (SD = 44.4, *n* = 25). The return in 16% of cases is by truck. In 54% of the cases, these pasture areas are communal land belonging to the municipalities, 43% communal land of the Regional Government and 3% private property. Communal pastures are normally hired by shepherds. The sheep herds share these areas with other sheep (57%, *n* = 25) or cattle (32%, *n* = 25) herds. The remaining 12% do not share the summer pastures with any other herd.

The grazing areas were not fenced, so grazing is rationed and guided by the shepherd, who controls livestock continuously, in 12–13 h shifts (92.0%, *n* = 25). When this herder is a contracted person (70% of the cases, *n* = 25), the farmer visits the livestock an average of 4–6 days a month to help in tasks such as herding, sanitary treatments or collecting waste. The two farmers who do not carry out this management (8.0%, *n* = 25) have herds of goats and leave them free on the pastures all day, checking their condition every 15 days. This handling is only possible when the pastures are not shared with other farmers. During the night, the herder collects and groups the livestock (80% of the cases, *n* = 25) with the help of meshes and electric fences (75%, *n* = 20) or in pens with fixed fences (25%, *n* = 20). Dogs work in livestock management in 96% of the farms (*n* = 25). Their mean number per herd is 3.0 (SD = 1.6, *n* = 24). For the protection of livestock, 54% of the farms (*n* = 24) have guard dogs (mastiffs), at an average of 2.5 per herd (SD = 1.0, *n* = 13). Modern technologies, such as the use of GPS for control, are not widely used (12.0%, *n* = 25). They point out that it is a very helpful tool as goats range freely all year round without supervision.

All the farmers say they take precautions to defend their sheep from predators, and they have their herds insured against possible attacks by them.

A total of 25 farmers consider that the available infrastructures in the mountain pastures are insufficient for extensive farming. Specifically, they consider it is necessary to improve: accesses (73.3%), shepherd’s shelters (46.6%), handling sleeves (43.5%), pens and fixed fences (38.1%), drinking troughs (26.8%), and mobile phone coverage (23.3%).

During the stay in the summer pastures, the sheep herds are usually grouped to facilitate management in the mountains and reduce labour with the consequent economic savings since many farmers hire a shepherd in the summer while the sheep are in the mountains. Conflicts between farmers on these pastures are minimal. Only two farmers have had problems concerning boundaries and grazing start dates.

### 3.4. Grazing in Natura 2000 Sites

A total of 60% (*n* = 30) of the farmers carry out their activity in Special Areas of Conservation of the Natura 2000 Network, and yet most of them (93.3%, *n* = 30) do not know what this protection status involves, the elements of the environment to be conserved, and the specific measures for the conservation of pastures, fauna and flora. The only two farmers who are aware of these measures have a very satisfactory opinion of them. The attitude to adapting their grazing activity to the requirements of the Natura 2000 network is high in 30% of the cases, low in 3.3% and indifferent in 66.6% (*n* = 30).

### 3.5. The Conflict with Wildlife and Dogs

All herders (*n* = 30) report conflicts with other animals during grazing. A value of 27% had conflicts with tourist dogs or hunting dogs running loose and disturbing the tranquillity of the herd or distracting the attention of the guard dogs; 60% with griffon vultures approaching lambing ewes or with sick animals lying motionless on the ground; 53% with wild boar which root pastures and meadows and break electric fences; 3.3% with roe deer breaking electric fences; 53% with a brown bear; and 33% with a wolf.

A total of 63% of all farmers have suffered direct damage caused by wildlife or predators. Farmers who have suffered damage from bears and wolves represent 23.3% and 10%, respectively. Up to 23% more expressed doubts about possible wolf attacks on their herds, arguing the disappearance of several heads of livestock at the end of the summer, although it could not be proven at the time. A total of 73% of the farmers claim to have a sporadic presence of bears in their grazing area. Regarding wolves, there is high uncertainty about their presence in the vicinity of their farms, even so, 46% confirm their presence.

Of the 19 farmers who reported predator damage, 79% of the cases were reported to the administration, of which only 26.6% (*n* = 15) received compensatory damages. These four cases were bear attacks.

Shepherds surveyed are taking several preventive measures to defend their sheep from predators. The most common are as follows: (i) removal of dead animals from the field where possible, (ii) guarding livestock at night during the lambing season, (iii) the use of predator-proof fencing at night, (iv) taking out private insurance for herds and (v) the use of guard dogs. All of the farmers consider mastiffs as a useful herd protection tool, and 56.6% own them for this purpose. Although they are absent in 13 of the farms, three of them stated that they were forced to give up their use due to the conflicts that arose in the summer pastures due to the interaction with tourist dogs who approached their herds.

### 3.6. Coexistence with Beasr and Wolves

All the farmers that have participated in this work have constant day and night surveillance of the herds by the shepherd during their stay in the mountains. Electric fences and the presence of mastiffs are the two prevention measures against predator attacks most used by farmers. If we compare the effectiveness of both measures according to the opinion of the farmers, they believe that the presence of mastiffs is much more effective in a possible attack scenario.

**Figure 2:**
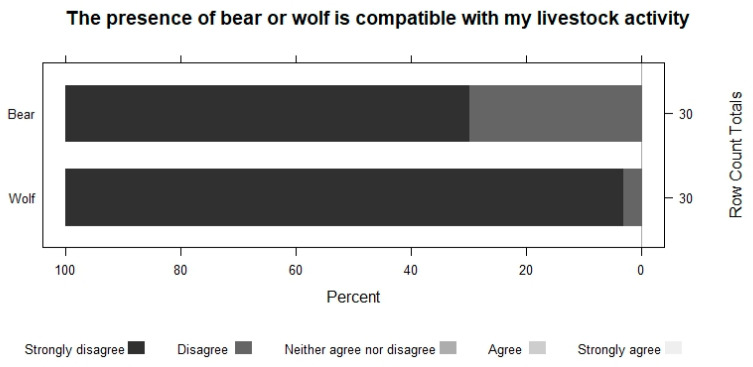
The compatibility of bears and wolves with livestock activity.

**Figure 3:**
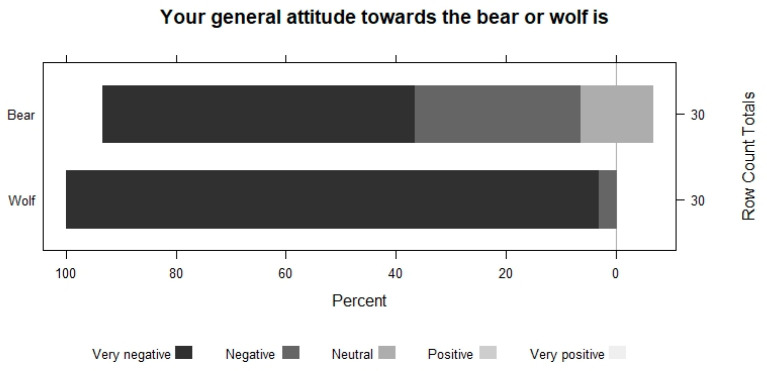
Shepherds’ attitude towards bears and wolves.

All the farmers who have participated in this work believe that the presence of wolves and extensive sheep farming are incompatible (Figure 2), and their attitude towards this species is very negative (Figure 3).

**Figure 4:**
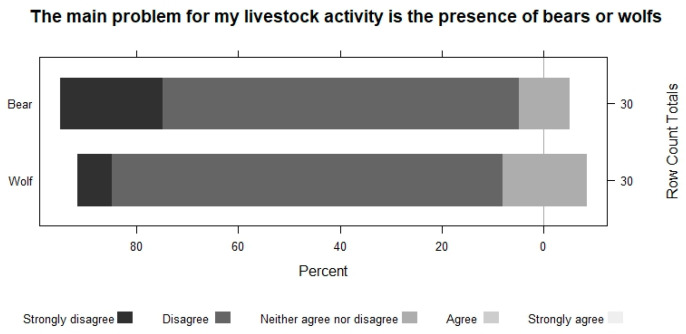
A consideration of the presence of bears and wolves as the main problem for extensive livestock farming.

Even with farmers presenting negative attitudes and a generalized rejection of these two species, they all share the opinion that the problems derived from bear or wolf attacks on herds are not the main problem faced by extensive livestock farming today (Figure 4).

Comparing the decisions that farmers would make in the face of a hypothetical scenario of a continuous bear or wolf presence in their grazing area, the presence of wolves would cause 76.6% (*n* = 30) of farmers to consider ceasing their activity. In the case of the bear, there would be greater tolerance, and 73.4% would continue to carry out their activity.

In case of having suffered an attack by a bear or a wolf to their herds, the proposal to receive compensation for every dead sheep, plus a fixed amount per head of flock attacked and annual agro-environmental aid would be accepted by 73.4% (*n* = 30) in the case of bear. In the case of the wolf, only 20% would accept the proposal. The remaining 80% believe that there is no possible financial compensation for such damage.

This more negative perception towards the wolf than the bear is also evident when farmers are asked about possible solutions to coexistence. A value of 70% of respondents (*n* = 30) think that the removal of wild animals from the area is the best solution, while for bears, only 20% chose this option. In the case of bears, the best coexistence option for farmers is more information from the administration on free-ranging individuals and their geolocalisation, which is demanded by 66.6% of those surveyed (*n* = 30). This option for the wolf is only accepted by 30% of the farmers.

A value of 77% believe that the presence of bears would not add economic and environmental value to the area, 16.6% do not know, and 6.8% think that they could be a tourist attraction (*n* = 30). In the case of the wolf, 100% agree that its presence in the mountains of the area would not add economic or environmental value.

**Figure 5:**
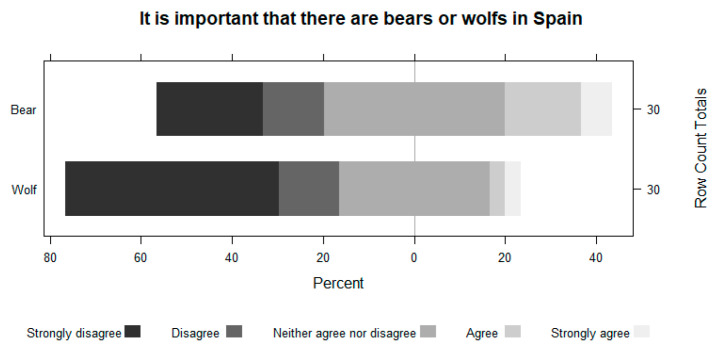
The importance of having bears and wolves in Spain.

**Figure 6:**
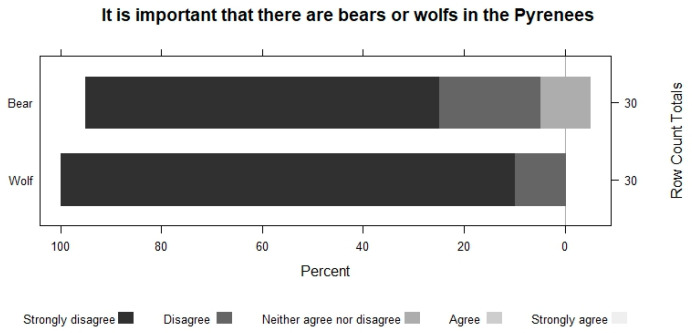
The Importance of having bears and wolves in the Pyrenees.

**Figure 7:**
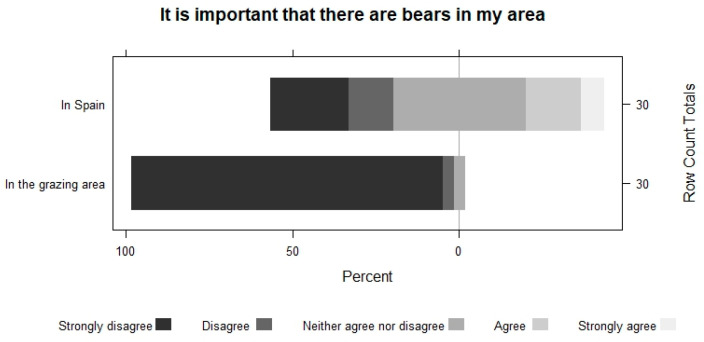
The importance of having bears in Spain and in the shepherds’ area.

**Figure 8:**
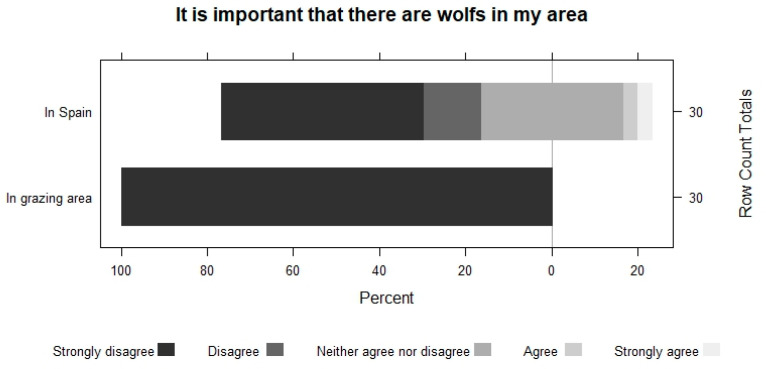
The importance of having wolves in Spain and in the shepherd’s area.

Farmers perceive that the importance of having bears and wolves decreases as they get closer to their area. They show the same pattern when asked about their conservation. For example, in the case of bears, 63.2% do not disagree with having bears in Spain, but 100% disagree with having them in the vicinity of their grazing area. The perception of wolves is always worse (Figure 5, Figure 6, Figure 7 and Figure 8).

## 4. Discussion

The interviewed shepherds preserve traditional management of their mountain farms, based on the use of forest, meadows, and forage crops around their villages and in the movement of the herds to summer pastures. This type of low-input management is responsible for the conservation of a large part of the biodiversity of European semi-natural habitats [1]. However, this activity is at risk of disappearing. In neighbouring valleys, the reduction of extensive sheep flocks since the 1980s is as high as 80% [15]. Grazing can be a tool to maintain or restore biodiversity in the open landscape and contributes to the aesthetic and recreational importance of grasslands. Their successful use for environmental protection and biodiversity enhancement requires careful planning and must be adapted to local conditions [16]. Pyrenean pastoralists possess extensive knowledge of the relationships between terrain, climate, vegetation, animal nutrition, and behaviour. Their disappearance leads to the loss of traditional ecological knowledge (TEK) [17].

Natura 2000 is a network of core breeding and resting sites for rare and threatened species and some rare natural habitat types, which are protected in their own right. It stretches across all 27 EU countries, both on land and at sea. The network aims to ensure the long-term survival of Europe’s most valuable and threatened species and habitats. (https://ec.europa.eu/environment/nature/natura2000/index_en.htm (accessed on 23 May 2023)). The majority of livestock farmers carry out their activity within this network, however, in this area, there is still no grazing management plan, no recognition for maintaining the habitats by grazing, and no subsidy line for European Common Agrarian Policy (CAP) associated with Natura 2000 site. There is a lack of voice in regional government decisions on local resources by farmers [17].

There is a generally negative perception of farmers towards bears and wolves. The view of bears is less negative, as it is perceived as part of mountain culture. During the carnival, people dress up as bears, not as wolves [18]. This difference is also common in situations where both species of large carnivores coincide with extensive sheep herds in Europe [19,20,21] and North America [22]. As both species are increasing in number and distribution in the Pyrenees, it must be considered that in Europe attitudes towards bears became more positive over time and became worse in the case of wolves the longer people coexisted with them [19]. In any case, monitoring changes in attitudes towards large carnivores over time is crucial [19,20] to adequately face the conflict, particularly in the case of extensive sheep farmers, who are the most affected because of livestock losses. All farmers considered that the presence of wolves in the mountains would not add economic or environmental value to the area, and many of them point out that not only would it not add value, but it would also be detrimental to sectors and activities that are currently of great importance in this area, such as mountain tourism, hunting, and mycology. They even consider that it would be a danger and a real threat to people’s safety.

The main problems of extensive sheep farmers are not bear and wolf attacks on their herds, as happens in other situations [11]. The crisis of the system is due to other problems, which resulted in a decrease in the number of sheep and farms [23]. Large carnivores are seen as a limitation to their livestock activities [24]. Coexistence measures will involve the adaptation of herders to new situations with changes in the use of grassland. For shepherds, the most desirable grazing areas were those with fewer terrain hazards, such as cliffs and talus slopes, minimizing the potential for animals to be killed or injured if they are spooked by a bear, wolf, or feral dog [17]. These factors will lead to changes in vegetation and loss of habitats by encroachment due to the abandonment of grazing in these areas [15].

Farmers stay with their herds permanently, unlike previous studies carried out in the Pyrenees [11] in which there were herds in the pastures without surveillance. An extensive sheep grazing system with little surveillance is believed not to be compatible with the existence of expanding brown bear populations [25]. For this reason, herds are less vulnerable to large carnivore attacks compared to other situations in the Pyrenees and GPS use is not generalized as it is not needed. Unfortunately, there is a lack of details on herd management, farms, and farmers in the international literature, regarding extensive sheep farms and their relation with large carnivores. This does not allow us to make proper comparisons and interpretations.

In the prevention of attacks by predators, fencing and electric mesh, mastiffs, and the presence of shepherds are essential. The use of guard dogs is considered a very effective method to reduce attacks on livestock in mountain environments [26]. Despite this, the application of protection measures against wolves in the Pyrenees and the Alps using guard dogs and electrified nocturnal pens has not given the expected result because wolves do not associate livestock with humans and humans with danger [27]. However, there are several experiences which show how to mitigate damages, including regular specialized publications (https://www.protectiondestroupeaux.ch/en/cdpnews/ (accessed on 23 May 2023)), even if scientific evidence is limited [28].

Livestock farmers are missing information from the administration on the presence of predators in the field to prevent attacks. In the French Pyrenees, for example, a tool has already been developed (https://info-ours.com/ (accessed on 23 May 2023)) for informing shepherds by SMS about the presence of the bear, with daily online information during the bear’s period of activity (March to November). The event sheet is updated every working day. It includes factual information on bear signs (footprints, hair, droppings, etc.), statements of damage, and their conclusion on the responsibility of the bear.

## 5. Conclusions

The farms are family-owned; in half of the cases, their continuity is not guaranteed. The extensive nature of the farms is manifested as a high degree of use of the mountain pastures during the summer and as continuous use during the rest of the year, based on meadows and fodder crops from the surroundings of the villages. The grazing practised is controlled 24 h a day by shepherds who unanimously complain about a lack of herding infrastructure in the mountain pastures to facilitate their work. The continuous presence of the shepherds with their herds justifies the scarce use of GPS collars. Farmers generally reject the presence of bears in the area and perceive the wolf as a real threat to the continuity of their economic activity, which presents a high degree of vulnerability. The compensations for damages are considered insufficient to convince them to coexist with the presence of bears and wolves. Prevention measures proposed are considered helpful tools but in no way as fully effective solutions.

## Data Availability

Data can be shared with other researchers upon request of collaboration.

## References

[1] O’rourke E., Charbonneau M., Poinsot Y. (2016). High nature value mountain farming systems in Europe: Case studies from the Atlantic Pyrenees, France and the Kerry Uplands, Ireland. J. Rural. Stud..

[2] Montserrat P., Fillat F. (2004). Pastos y Ganadería Extensiva. Evolución Reciente de la Ganadería Extensiva Española y Perspectivas. http://hdl.handle.net/10261/100210.

[3] Manrique E., Zamudio A., Olaizola A. (2006). The Economic Effects of the CAP Reform on Aragonese Sheep Farms. 2° Seminario de la Red Científico-Profesional de Ganadería Mediterránea. Las Producciones Ganaderas Mediterráneas: Incertidumbres y Oportunidades. Zaragoza, Centro Internacional de Altos Estudios Agronómicos Mediterráneos. http://om.ciheam.org/article.php?IDPDF=800253.

[4] Olaizola A. (1991). Viabilidad Económica de Sistemas Ganaderos de Montaña en Condiciones de Competencia en el Uso de Factores Productivos. Análisis de la Ganadería en un Valle Pirenaico Característico Mediante Técnicas Multivariantes y de Optimización. Ph.D. Thesis.

[5] Hovardas T. (2018). Large Carnivore Conservation and Management: Human Dimensions.

[6] Milheiras S.G., Hodge I. (2011). Attitudes towards compensation for wolf damage to livestock in Viana do Castelo, North of Portugal. Innov. Eur. J. Soc. Sci. Res..

[7] Linnell J.D., Odden J., Smith M.E., Aanes R., Swenson J.E. (1999). Large carnivores that kill livestock: Do “problem individuals” really exist?. Wildl. Soc. Bull..

[8] Karlsson J., Johansson Ö. (2010). Predictability of repeated carnivore attacks on livestock favours reactive use of mitigation measures. J. Appl. Ecol..

[9] Quenette P., Alonso M., Chayron L., Cluzel P., Dubarry E., Dubreuil D., Palazon S., Pomarol M. (2001). Preliminary results of the first transplantation of brown bears in the French Pyrenees. Ursus.

[10] Palazón S., Catalan J., Ninot J.M., Aniz M.M. (2017). The Importance of Reintroducing Large Carnivores: The Brown Bear in the Pyrenees. High Mountain Conservation in a Changing World.

[11] Herrero J., García-Serrano A., Reiné R., Ferrer V., Azón R., López-Bao J.V., Palomero G. (2021). Challenges for recovery of large carnivores in humanized countries: Attitudes and knowledge of sheep farmers towards brown bear in Western Pyrenees, Spain. Eur. J. Wildl. Res..

[12] Instituto Aragonés de Estadística Distribución Comarcal Ganadería 2020. https://www.aragon.es/buscador?type=com.liferay.journal.model.JournalArticle&type=es.aragon.sede.service.model.Service&type=es.aragon.sede.service.model.Child&q=Di.

[13] Manrique E., Revilla R., Saez E. (1987). Características Estructurales del Sector Agroganadero de la Comarca de Ribagorza.

[14] Ferrer C. (2016). Diccionario de Pascología. Aspectos Ecológicos, Botánicos, Agronómicos, Forestales, Zootécnicos y Socioeconómicos de los Pastos.

[15] Gartzia M., Fillat F., Pérez-Cabello F., Alados C.L. (2016). Influence of Agropastoral System Components on Mountain Grassland Vulnerability Estimated by Connectivity Loss. PLoS ONE.

[16] Metera E., Sakowski T., Sloniewski K., Romanowicz B. (2010). Grazing as a tool to maintain biodiversity of grassland—A review. Anim. Sci. Pap. Rep..

[17] Fernández-Giménez M.E., Estaque F.F. (2012). Pyrenean Pastoralists’ Ecological Knowledge: Documentation and Application to Natural Resource Management and Adaptation. Hum. Ecol..

[18] Bergua Amores J.A. (2011). El conflicto ocasionado por la introducción de osos en los Pirineos. Diferentes interpretaciones de los contratos natural y nacional. Rev. Int. De Sociol..

[19] Klenzendorf S.A., Vaughan M.R. (1999). An overview of brown bear management in six European countries. Ursus.

[20] Dressel S., Sandström C., Ericsson G. (2015). A meta-analysis of studies on attitudes toward bears and wolves across Europe 1976–2012. Conserv. Biol..

[21] Franchini M., Corazzin M., Bovolenta S., Filacorda S. (2021). The return of Large Carnivores and Extensive Farming Systems: A Review of Stakeholders’ Perception at an EU Level. Animals.

[22] Bangs E., Jimenez M., Niemeyer C., Fontaine J., Collinge M., Krsichke R., Handegard L., John A.S., Sime C., Nadeau S. (2006). Non-lethal and lethal tools to manage wolf-livestock conflict in the Northwestern United States. Proc. Vertebr. Pest Conf..

[23] Roldán L. (2016). El ovino y el Caprino En Aragón. Evolución en los 20 Últimos Años (1996–2016).

[24] Bisi J., Liukkonen T., Mykrä S., Pohja-Mykrä M., Kurki S. (2010). The good bad wolf—wolf evaluation reveals the roots of the Finnish wolf conflict. Eur. J. Wildl. Res..

[25] Sagør J.T., Swenson J.E., Røskaft E. (1997). Compatibility of brown bear Ursus arctos and free-ranging sheep in Norway. Biol. Conserv..

[26] Landry J.-M., Borelli J.-L., Drouilly M. (2020). Interactions between livestock guarding dogs and wolves in the southern French Alps. J. Vertebr. Biol..

[27] Meuret M., Moulin C.-H., Bonnet O., Garde L., Nozieres-Petit M.-O., Lescureux N. (2021). Missing shots: Has the possibility of shooting wolves been lacking for 20 years in France’s livestock protection measures?. Rangel. J..

[28] Eklund A., López-Bao J.V., Tourani M., Chapron G., Frank J. (2017). Limited evidence on the effectiveness of interventions to reduce livestock predation by large carnivores. Sci. Rep..

